# β-Hydroxybutyrate Modulates Metabolic Signaling and Partially Restores Peripheral Circadian Rhythms in High-Fat Diet-Fed Mice

**DOI:** 10.3390/foods15081305

**Published:** 2026-04-09

**Authors:** Natalie Avital-Cohen, Nava Chapnik, Oren Froy

**Affiliations:** Institute of Biochemistry, Food Science and Nutrition, Robert H. Smith Faculty of Agriculture, Food and Environment, The Hebrew University of Jerusalem, Rehovot 76100, Israel; natalie.avital@mail.huji.ac.il (N.A.-C.); nava.chapnik@mail.huji.ac.il (N.C.)

**Keywords:** β-hydroxybutyrate, circadian rhythms, high-fat diet, obesity, metabolism, muscle, adipose tissue

## Abstract

A high-fat (HF) diet disrupts metabolic homeostasis and impairs peripheral circadian rhythms in key metabolic tissues. β-Hydroxybutyrate (BHB), a major circulating ketone body, functions not only as an energy substrate but also as a signaling metabolite regulating nutrient-sensing and inflammatory pathways. However, its role in modulating metabolic–circadian interactions under conditions of nutrient excess remains unclear. In this study, we investigated whether BHB supplementation influences metabolic signaling and circadian clock oscillations in liver, skeletal muscle and adipose tissue under chow and HF conditions. Male C57BL/6 mice were fed chow or HF with or without BHB supplementation (500 mg/kg body weight in the diet) for 7 weeks. Metabolic parameters were assessed by indirect calorimetry, and tissues were collected every 4 h across the circadian cycle. HF feeding increased body weight and adiposity (*p* < 0.01), reduced AMPK activation, enhanced AKT/mTOR signaling, elevated NF-κB levels and dampened clock gene rhythmicity. BHB supplementation significantly decreased food intake in HF-fed mice (*p* < 0.01) and partially reversed several molecular alterations in a tissue-specific manner. In skeletal muscle and adipose tissue, BHB increased AMPK activation and reduced mTOR and NF-κB signaling (*p* < 0.05), whereas hepatic effects were more modest. Notably, BHB modulated circadian gene expression, restoring aspects of rhythmic amplitude and/or phase, particularly in adipose tissue. These findings may indicate that BHB supplementation modulates nutrient-sensing pathways and partially restores peripheral circadian rhythms under HF conditions. While some effects may be influenced by reduced energy intake, BHB may serve as a metabolic signal linking nutrient status to circadian regulation.

## 1. Introduction

Circadian rhythms are endogenous, approximately 24-h oscillations that coordinate behavior and physiology with the external light–dark cycle. In mammals, these rhythms are orchestrated by a central pacemaker located in the suprachiasmatic nucleus (SCN) of the hypothalamus and by peripheral clocks present in nearly all tissues, including liver, adipose tissue and skeletal muscle [1]. At the molecular level, circadian clocks are driven by interconnected transcription–translation feedback loops involving core clock components, such as Brain and Muscle ARNT-Like 1 (BMAL1), Circadian Locomotor Output Cycles Kaput (CLOCK), Period (PER) and Cryptochrome (CRY) proteins [2]. These molecular oscillators regulate a large fraction of the transcriptome in metabolic tissues, thereby temporally coordinating pathways involved in glucose homeostasis, lipid metabolism, mitochondrial function and energy expenditure [3].

Metabolic homeostasis is tightly linked to circadian timing. Disruption of circadian rhythms through genetic manipulation, shift work, or altered feeding schedules predisposes to obesity, insulin resistance, dyslipidemia and nonalcoholic fatty liver disease [4]. Conversely, nutritional cues act as potent zeitgebers for peripheral clocks. Feeding time, caloric intake and macronutrient composition can reset or modulate clock gene expression in metabolic tissues independently of the SCN [5]. High-fat feeding, in particular, has been shown to dampen circadian amplitude, alter rhythmic gene expression, and impair metabolic flexibility [6,7].

Ketone bodies are metabolic substrates produced primarily in the liver during periods of low carbohydrate availability, prolonged fasting, or ketogenic diets [8]. β-Hydroxybutyrate (BHB) is the most abundant circulating ketone body and serves not only as an alternative energy source but also as a signaling metabolite [9]. Beyond its classical bioenergetic role, BHB modulates cellular signaling pathways, including AMP-activated protein kinase (AMPK), mammalian target of rapamycin (mTOR), and insulin/AKT signaling, all of which are central regulators of nutrient sensing and metabolic adaptation [9,10]. Moreover, BHB has been shown to inhibit class I histone deacetylases (HDACs), thereby influencing gene expression through epigenetic mechanisms [9,11].

Emerging evidence indicates that ketone bodies may interact with the circadian system [12]. Nutrient-sensing pathways such as AMPK and mTOR directly regulate core clock components, including BMAL1 and CRY1, linking cellular energy status to clock function [5,13,14]. Additionally, fasting, which robustly elevates circulating BHB, alters peripheral clock gene expression and phase [15,16]. However, whether BHB itself directly modulates circadian oscillations in metabolic tissues under conditions of nutrient excess remains poorly understood. Given that BHB exerts anti-inflammatory effects through inhibition of the NLRP3 inflammasome [17,18] and regulates key metabolic signaling cascades, it is plausible that BHB supplementation could modulate circadian and metabolic pathways, particularly in the context of diet-induced metabolic stress.

Importantly, several of the above-mentioned pathways directly interact with the molecular circadian clock. AMPK promotes the degradation of CRY proteins, thereby resetting clock timing, whereas mTOR signaling has been implicated in the regulation of circadian translation and rhythmic protein synthesis. In addition, epigenetic regulation via HDAC inhibition by BHB may influence transcription of core clock genes. These interactions suggest a mechanistic framework through which BHB could modulate circadian rhythms via metabolic signaling pathways.

Despite growing evidence linking ketone bodies to metabolic signaling and circadian regulation, it remains unclear whether BHB directly modulates peripheral clock function under conditions of nutrient excess, such as high-fat diet feeding. In particular, the extent to which BHB influences the amplitude, phase, and tissue-specific coordination of circadian rhythms alongside metabolic signaling pathways has not been systematically examined. Therefore, the aim of this study was to determine whether BHB supplementation modulates metabolic signaling pathways and circadian clock oscillations in liver, skeletal muscle and adipose tissue under chow and high-fat diet conditions. Understanding the interplay between ketone body signaling, dietary composition and circadian regulation may reveal novel mechanisms linking metabolic state to temporal control of physiology.

## 2. Materials and Methods

### 2.1. Animals, Treatments and Tissue Collection

Ninety-six 9-week-old male C57BL/6 mice were housed in a temperature- and humidity-controlled facility (23–24 °C, 60% humidity) under a 12 h light/12 h dark cycle (12:12 LD). Animals were acclimated for 10 days with ad libitum (AL) access to food and water. Following acclimation, mice were randomly assigned to one of four experimental groups (*n* = 24 per group): Chow, Chow + BHB, high-fat diet (HF), and HF + BHB. BHB was incorporated into the diet at a dose of 500 mg/kg body weight, based on our previous study with a very similar molecule, β-hydroxymethyl butyrate, demonstrating metabolic signaling effects at this range [19]. Animals were maintained on their respective diets for 7 weeks with ad libitum access to food. At the end of the experimental period, circadian sampling was performed over a 24 h cycle. Mice were transferred to constant darkness (DD) conditions prior to tissue collection, and all procedures were conducted under dim red light to avoid light-induced circadian disruption. Samples were collected at six evenly spaced time points (every 4 h), with 3–4 mice per group at each time point. At each time point, mice were anesthetized using isoflurane, and blood samples were collected, followed by rapid dissection of liver, epididymal adipose tissue, and gastrocnemius muscle. Tissues were immediately frozen in liquid nitrogen and stored at −80 °C until further analysis. Mice were subsequently euthanized following tissue collection. Fasting blood β-hydroxybutyrate (β-HB) levels were measured using a glucometer (Optium Xceed; Abbott Laboratories, Maidenhead, UK).

### 2.2. Ethical Approval

All experimental procedures were conducted in accordance with the Guide for the Care and Use of Laboratory Animals (NIH publication 86-23, revised 1985). The study was approved by the Institutional Animal Care and Use Committee (IACUC) of the Hebrew University and Hadassah Medical Center (approval number: AG-20-16417-4).

### 2.3. Assessment of Metabolic Parameters

Indirect calorimetry, energy expenditure, locomotion and activity were measured using the Promethion Metabolic Cage System (Sable Systems, North Las Vegas, NV, USA). Mice were habituated to the metabolic cages for 3 days prior to data collection. Water was available throughout the study and lighting conditions matched those of the home cages. Respiratory gases were analyzed using an integrated fuel-cell oxygen analyzer and a spectrophotometric carbon dioxide analyzer. Oxygen consumption (VO_2_) and carbon dioxide production (VCO_2_) were calculated in mL/min. The respiratory exchange ratio (RER) was determined as VCO_2_/VO_2_. Locomotor activity was quantified as the sum of all directed ambulatory movements ≥1 cm/s, detected using a three-dimensional (x, y, z) infrared beam-break system.

### 2.4. Diet Composition

The control diet consisted of a standard low-fat (LF) chow containing 53% cornstarch, 20% casein, 10% sucrose, 7% soybean oil, 5% cellulose, 4% mineral mix, 1% vitamin mix and 0.3% methionine. The HF diet contained 38% cornstarch, 20% casein, 10% sucrose, 7% soybean oil, 15% coconut oil, 5% cellulose, 4% mineral mix, 1% vitamin mix and 0.3% methionine. Overall, the HF diet provided 22% fat by weight (42% kcal from fat), primarily derived from soybean oil and coconut oil, compared with 7% fat by weight (16% kcal from fat) in the chow diet. The caloric density of the HF diet was 4.7 kcal/g versus 3.95 kcal/g for the chow diet. Mice in the BHB-supplemented groups received 500 mg BHB per kg body weight.

### 2.5. Serum Analyses and ELISA

Blood was collected from the inferior vena cava at sacrifice. Samples were allowed to clot at room temperature for 30 min and then centrifuged at 2000× *g* for 15 min. Serum was collected, frozen and stored at −20 °C until analysis. Serum alanine aminotransferase (ALT/SGPT), aspartate aminotransferase (AST/SGOT), HDL, total cholesterol, and triglycerides were measured using ELISA (American Medical Laboratories, Herzliya, Israel).

### 2.6. Western Blot Analysis

Tissues were homogenized in 200 μL lysis buffer (pH 7.8) containing 20 mM Tris, 145 mM NaCl, 5% glycerol, 1% Triton X-100, 50 nM PMSF, 50 μM NaF, 10 μM Na_3_VO_4_, 50 ng/mL aprotinin, 100 ng/mL leupeptin and 0.8 μg/mL trypsin inhibitor (Sigma, Rehovot, Israel). Protein samples were separated on 12% SDS-polyacrylamide gels and semi-dry transferred onto nitrocellulose membranes. Membranes were incubated with primary antibodies against AMPK and pAMPK, BMAL1 and pBMAL1, AKT and pAKT, P70S6K and pP70S6K, S6 and pS6, ACC and pACC, fatty acid synthase (FASN) (Cell Signaling Technology, Danvers, MA, USA), ACTIN, mTOR, MYOGENIN, CLOCK, CRY1 and NF-κB (Santa Cruz Biotechnology, Dallas, TX, USA). After washing, membranes were incubated with horseradish peroxidase-conjugated secondary antibodies (Pierce, Rockford, IL, USA). Actin was used as a loading control and detected with an anti-mouse antibody (MP Biomedicals, Solon, OH, USA). Immunoreactive bands were visualized by enhanced chemiluminescence and quantified by densitometry. Protein expression levels were expressed in arbitrary units.

### 2.7. RNA Extraction and Quantitative Real-Time PCR

Total RNA was extracted using TRI Reagent (Sigma, Rehovot, Israel). RNA samples were treated with RQ1 DNase (Promega, Madison, WI, USA) and reverse-transcribed using the qScript cDNA synthesis kit (Quanta BioSciences, Gaithersburg, MD, USA) with random hexamers (Promega). Quantitative real-time PCR was performed using exon-exon spanning primers [20] and the ABI Prism 7300 Sequence Detection System (Applied Biosystems, Foster City, CA, USA). Gene expression was normalized to actin. PCR conditions were as follows: 3 min at 95 °C, followed by cycles of 10 s at 95 °C and 45 s at 60 °C. Relative gene expression was calculated using the 2^−ΔΔCt^ method.

### 2.8. Statistical Analysis

Data are presented as mean ± SE. Differences in average protein expression were analyzed using Student’s *t*-test or Tukey’s honestly significant difference (HSD) test, as appropriate. Circadian and group differences were analyzed using two-way ANOVA (diet × time), followed by Tukey’s post hoc test where appropriate. One-way ANOVA was used for single-factor comparisons. Circadian patterns across multiple time points were analyzed using one-way ANOVA (time of day). Statistical significance was set at *p* < 0.05. Analyses were performed using JMP Pro 18 (SAS Institute Inc., Cary, NC, USA). Circadian rhythmicity (amplitude, phase, and mesor) was further analyzed using CircWave software (version 1.4; Circadian Rhythm Laboratory, University of Groningen, The Netherlands) by applying harmonic regression or Fourier-curve fit analyses to biological data.

## 3. Results

### 3.1. Effect of BHB on Weight, Metabolism and Serum Parameters

**Figure 1:**
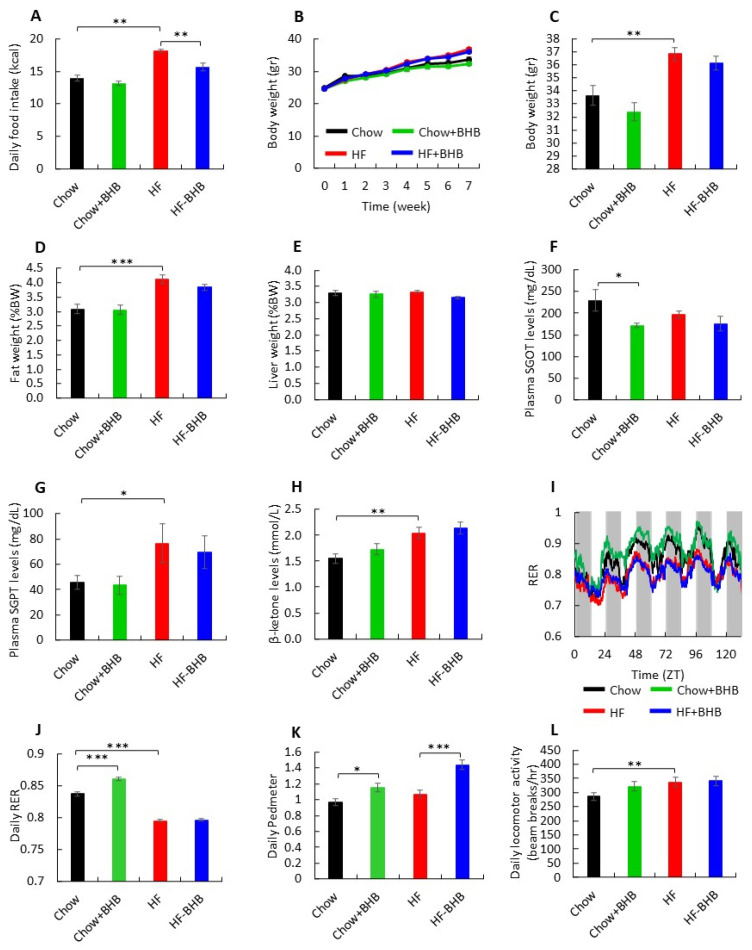
Effect of BHB on serum parameters, whole-body physiology and activity. (**A**) Body weight during the experiment. (**B**) Final body weight. (**C**) Daily food intake. (**D**) Fat tissue weight. (**E**) Liver weight. (**F**) Plasma SGOT. (**G**) Plasma SGPT. (**H**) β-ketone levels. (**I**) Respiratory exchange ratio (RER). (**J**) Average daily respiratory exchange ratio (RER). (**K**) Mean daily distance. (**L**) Daily locomotor activity. Data are presented as mean ± SEM. * *p* < 0.05, ** *p* < 0.01, *** *p* < 0.0001.

Mice fed a high-fat (HF) diet exhibited a marked increase in daily food intake compared with Chow animals (*p* < 0.0001) (Figure 1A). Supplementation with β-hydroxybutyrate (BHB) significantly reduced food intake in HF-fed mice compared with HF animals (*p* < 0.01) (Figure 1A). Body weight increased progressively over the 7-week experimental period in all groups; however, HF-fed mice displayed significantly higher body weights than Chow-fed mice beginning at week 5. By the end of the study, both HF and HF + BHB groups had significantly greater body weights than Chow-fed mice and Chow + BHB mice (*p* < 0.01) (Figure 1B,C). Consistent with increased adiposity, fat mass (expressed as a percentage of body weight) was significantly elevated in HF-fed mice compared with Chow-fed mice (*p* < 0.0001) (Figure 1D), whereas liver weight relative to body weight did not differ among groups (Figure 1E). Liver enzyme AST/SGOT (aspartate aminotransferase) levels in chow group decreased after BHB supplementation compared with Chow animals (*p* < 0.05) (Figure 1F).

ALT/SGPT (alanine aminotransferase) levels were significantly elevated in HF-fed mice compared with Chow-fed mice (*p* < 0.05) (Figure 1G). Serum cholesterol, HDL-cholesterol and triglycerides did not differ among all groups. Circulating ketone levels were higher in the HF group compared with Chow animals (*p* < 0.01) (Figure 1H), although no differences were detected between Chow-fed mice vs. Chow+BHB or HF vs. HF + BHB groups. Assessment of energy metabolism revealed clear diurnal fluctuations in respiratory exchange ratio (RER) across all groups (Figure 1I). The average daily RER was significantly higher in Chow + BHB mice than in Chow-fed mice, whereas both HF and HF + BHB groups exhibited significantly lower RER values (*p* < 0.0001) (Figure 1J). Physical activity measurements further showed increased daily pedometer activity in both BHB-treated groups relative to their respective Chow- or HF-fed mice (*p* < 0.05 and *p* < 0.0001, respectively) (Figure 1K). Total locomotor activity (beam breaks/h) was also significantly higher in HF-fed mice compared with Chow-fed mice (Figure 1L).

### 3.2. Effect of BHB on Liver Metabolism

**Figure 2:**
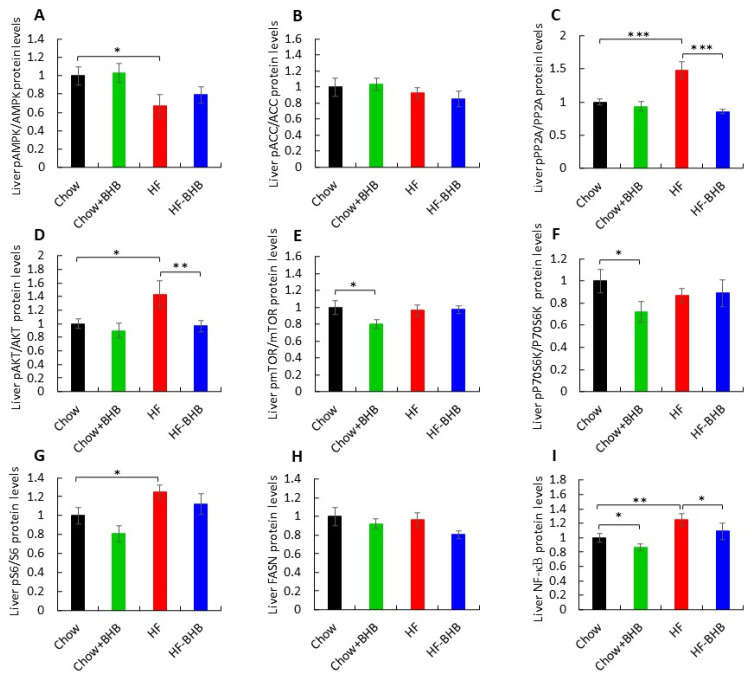
Effect of BHB on liver metabolic signaling. (**A**) Average daily pAMPK/AMPK levels in liver tissue. (**B**) Average daily pACC/ACC levels in liver tissue. (**C**) Average daily pPP2A/PP2A levels in liver tissue. (**D**) Average daily pAKT/AKT levels in liver tissue. (**E**) Average daily pmTOR/mTOR1 levels in liver tissue. (**F**) Average daily pP70/P70 levels in liver tissue. (**G**) Average daily pS6/s6 levels in liver tissue. (**H**) Average daily FASN levels in liver tissue. (**I**) Average daily NF-κB levels in liver tissue. Data are presented as mean ± SEM. * *p* < 0.05, ** *p* < 0.01, *** *p* < 0.0001.

At the molecular level, HF feeding reduced hepatic AMPK activation, as reflected by a lower pAMPK/AMPK ratio compared with Chow-fed mice (*p* < 0.05) (Figure 2A, Supplementary Figure S1). In contrast, no significant differences were observed in pACC/ACC ratios among groups (Figure 2B, Supplementary Figure S1).

HF feeding significantly increased hepatic pPP2A/PP2A and pAKT/AKT ratios, an effect that was markedly attenuated by BHB supplementation in HF-fed mice (*p* < 0.05) (Figure 2C,D, Supplementary Figure S1). In Chow-fed mice, BHB supplementation significantly reduced the pmTOR/mTOR and pP70S6K/P70S6K ratios (*p* < 0.05) (Figure 2E,F, Supplementary Figure S1), whereas pS6/S6 ratios remained unchanged (Figure 2G, Supplementary Figure S1). Fatty acid synthase (FASN) protein levels did not differ among groups (*p* > 0.05) (Figure 2H, Supplementary Figure S1). Inflammatory signaling was also affected. NF-κB protein levels were significantly reduced in Chow + BHB mice compared with Chow-fed mice (*p* < 0.05) (Figure 2I, Supplementary Figure S1), whereas HF feeding markedly increased NF-κB expression compared with Chow animals (*p* < 0.01) (Figure 2I, Supplementary Figure S1). Importantly, BHB supplementation reversed this HF-induced increase (*p* < 0.05) (Figure 2I, Supplementary Figure S1) in the liver.

### 3.3. Effect of BHB on Muscle Metabolism

**Figure 3:**
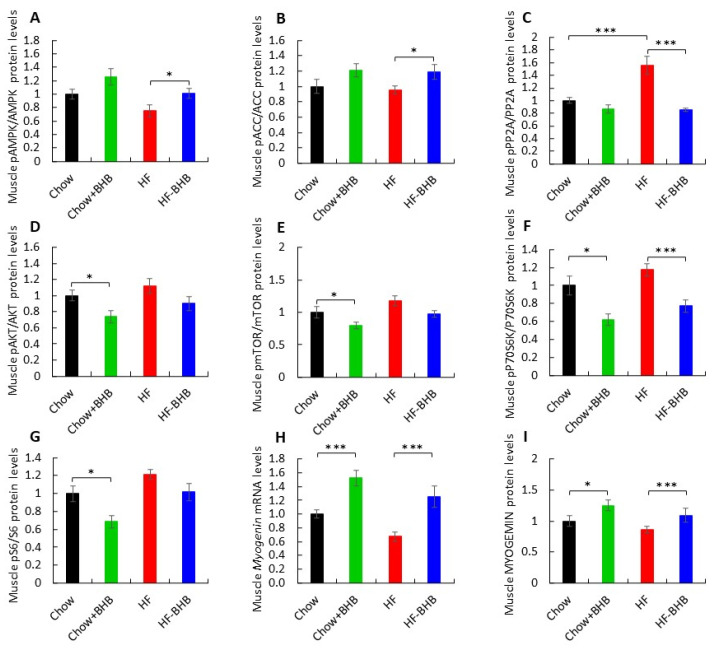
Effect of BHB on muscle metabolism. (**A**) Average daily pAMPK/AMPK levels in muscle tissue. (**B**) Average daily pACC/ACC levels in muscle tissue. (**C**) Average daily pPP2A/PP2A levels in muscle tissue. (**D**) Average daily pAKT/AKT levels in muscle tissue. (**E**) Average daily pmTOR/mTOR1 levels in muscle tissue. (**F**) Average daily pP70/P70 levels in muscle tissue. (**G**) Average daily pS6/s6 levels in muscle tissue. (**H**) Average daily *Myogenin* mRNA levels in muscle tissue. (**I**) Average daily MYOGENIN levels in muscle tissue. Data are presented as mean ± SEM. * *p* < 0.05, *** *p* < 0.0001.

In skeletal muscle, BHB supplementation significantly increased the pAMPK/AMPK ratio in HF-fed mice compared with the HF group (*p* < 0.05) (Figure 3A, Supplementary Figure S2). A similar pattern was observed for pACC/ACC (*p* < 0.05) (Figure 3B), while pPP2A/PP2A levels were markedly reduced (*p* < 0.0001) (Figure 3C, Supplementary Figure S2), collectively indicating enhanced metabolic activation.

Conversely, anabolic signaling appeared suppressed by BHB. Muscle pAKT/AKT levels were lower in Chow + BHB mice than in Chow-fed mice (*p* < 0.05) (Figure 3D, Supplementary Figure S2). Consistently, pmTOR/mTOR and pP70S6K/P70S6K and pS6/S6 ratios were reduced in BHB-treated Chow-fed groups compared with Chow animals (*p* < 0.05, *p* < 0.01 and *p* < 0.05, respectively) (Figure 3E–G, Supplementary Figure S2), and the pP70S6K/P70S6K ratio was also reduced in HF-fed mice treated with BHB (*p* < 0.05) (Figure 3F, Supplementary Figure S2). Notably, muscle *Myogenin* mRNA and protein expression were significantly elevated in both Chow + BHB and HF + BHB groups (*p* < 0.0001) (Figure 3H,I, Supplementary Figure S2), indicating enhanced myogenic signaling in response to BHB supplementation.

### 3.4. Effect of BHB on Adipose Tissue Metabolism

**Figure 4:**
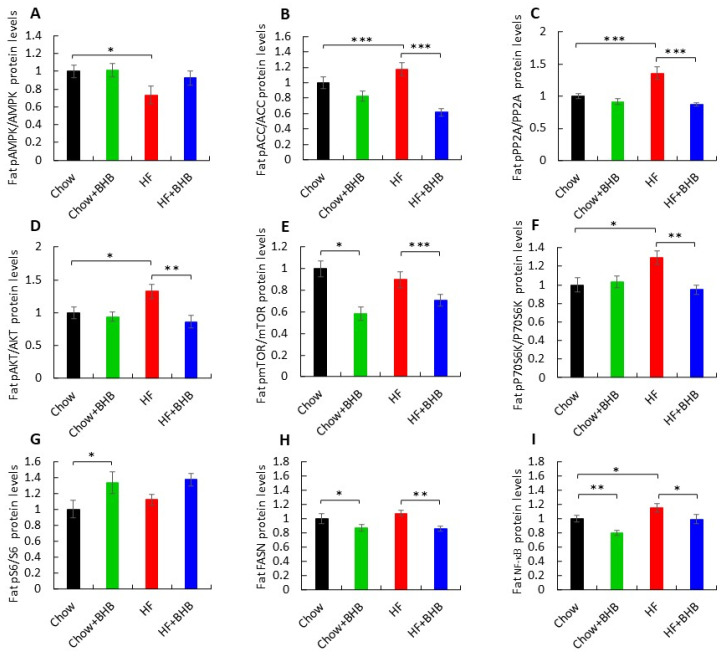
Effect of BHB on adipose tissue metabolism. (**A**) Average daily pAMPK/AMPK levels in adipose tissue. (**B**) Average daily pACC/ACC levels in adipose tissue. (**C**) Average daily pPP2A/PP2A levels in adipose tissue. (**D**) Average daily pAKT/AKT levels in adipose tissue. (**E**) Average daily pmTOR/mTOR1 levels in adipose tissue. (**F**) Average daily pP70/P70 levels in adipose tissue. (**G**) Average daily pS6/s6 levels in adipose tissue. (**H**) Average daily FASN levels in adipose tissue. (**I**) Average daily NF-κB levels in adipose tissue. Data are presented as mean ± SEM. * *p* < 0.05, ** *p* < 0.01, *** *p* < 0.0001.

In adipose tissue, HF feeding significantly reduced the pAMPK/AMPK ratio compared with Chow-fed mice, whereas BHB supplementation restored AMPK activation in HF-fed mice (*p* < 0.05) (Figure 4A, Supplementary Figure S3). HF feeding increased pACC/ACC and pPP2A/PP2A ratios, both of which were reduced by BHB supplementation (*p* < 0.0001) (Figure 4B,C, Supplementary Figure S3). A similar pattern was observed for pAKT/AKT levels (*p* < 0.05 and *p* < 0.01, respectively) (Figure 4D, Supplementary Figure S3). BHB supplementation reduced the pmTOR/mTOR ratio under both chow and HF conditions (*p* < 0.0001 and *p* < 0.05, respectively) (Figure 4E, Supplementary Figure S3).

Likewise, the HF-induced increase in pP70S6K/P70S6K was attenuated by BHB (*p* < 0.01) (Figure 4F, Supplementary Figure S3). The pS6/S6 ratio was increased in Chow + BHB mice (*p* < 0.05) (Figure 4G, Supplementary Figure S3). FASN protein levels were reduced by BHB supplementation irrespective of diet (*p* < 0.05 and *p* < 0.01) (Figure 4H, Supplementary Figure S3). In parallel, NF-κB levels were elevated by HF feeding compared with Chow animals (*p* < 0.05) and significantly reduced by BHB treatment under both dietary conditions (*p* < 0.01) (Figure 4I, Supplementary Figure S3), supporting an anti-inflammatory role for BHB in adipose tissue.

### 3.5. Effect of BHB on Liver Circadian Rhythms

**Figure 5:**
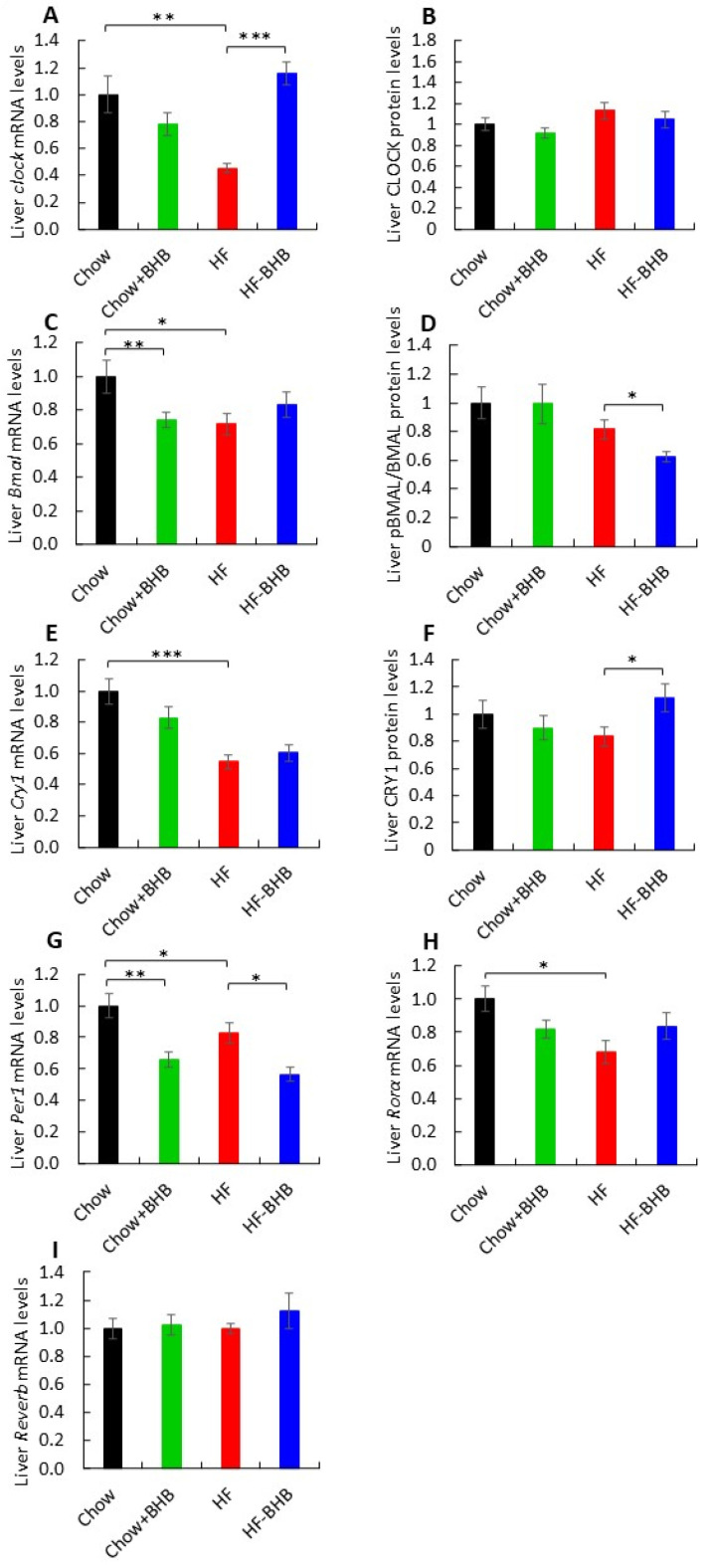
Effect of BHB on liver circadian rhythms. (**A**) Average daily *Clock* mRNA levels in liver tissue. (**B**) Average daily CLOCK levels in liver tissue. (**C**) Average daily *Bmal1* mRNA levels in liver tissue. (**D**) Average daily BMAL1 levels in liver tissue. (**E**) Average daily *Cry1* mRNA levels in liver tissue. *(***F**) Average daily CRY1 levels in liver tissue. (**G**) Average daily *Per1* mRNA levels in liver tissue. (**H**) Average daily *Rorα* mRNA levels in liver tissue. (**I**) Average daily *Rev-erbα* mRNA levels in liver tissue. Circadian parameters (amplitude, phase) were derived using CircWave harmonic regression analysis. Data are presented as mean ± SEM. * *p* < 0.05, ** *p* < 0.01, *** *p* < 0.0001.

HF feeding markedly disrupted hepatic circadian gene expression. *Clock* mRNA, but not protein levels, were significantly reduced in HF mice compared with Chow-fed mice and HF + BHB mice (*p* < 0.01 and *p* < 0.0001, respectively) (Figure 5A,B, Supplementary Figure S4), and rhythmicity was substantially blunted. BHB supplementation partially restored rhythmicity and induced a phase delay (Supplementary Figure S5A). Similarly, *Bmal1* mRNA exhibited diurnal oscillation and was significantly higher only in Chow animals (Figure 5C, Supplementary Figure S5B), while the pBMAL1/BMAL1 ratio remained unaffected and was reduced only in the HF + BHB group (Figure 5D, Supplementary Figure S4).

*Cry1* mRNA levels were decreased in HF mice compared with Chow animals (*p* < 0.0001) (Figure 5E), yet CRY1 protein levels were increased in HF + BHB animals compared with HF animals (*p* < 0.01) (Figure 5F, Supplementary Figure S4). *Cry1* expression retained rhythmicity, with BHB inducing a phase delay (Supplementary Figure S5C). *Per1* mRNA expression was reduced by BHB compared with Chow or HF animals (*p* < 0.0001) (Figure 5G) but displayed increased oscillation amplitude (Supplementary Figure S5D). *Rorα* expression was lower in HF mice compared with Chow animals (*p* < 0.05) (Figure 5H) yet remained rhythmic (Supplementary Figure S5E). *Rev-erbα* expression did not differ significantly among groups (Figure 5I) but maintained a diurnal pattern, with higher peak expression in HF mice (Supplementary Figure S5F).

### 3.6. Effect of BHB on Muscle Circadian Rhythms

**Figure 6:**
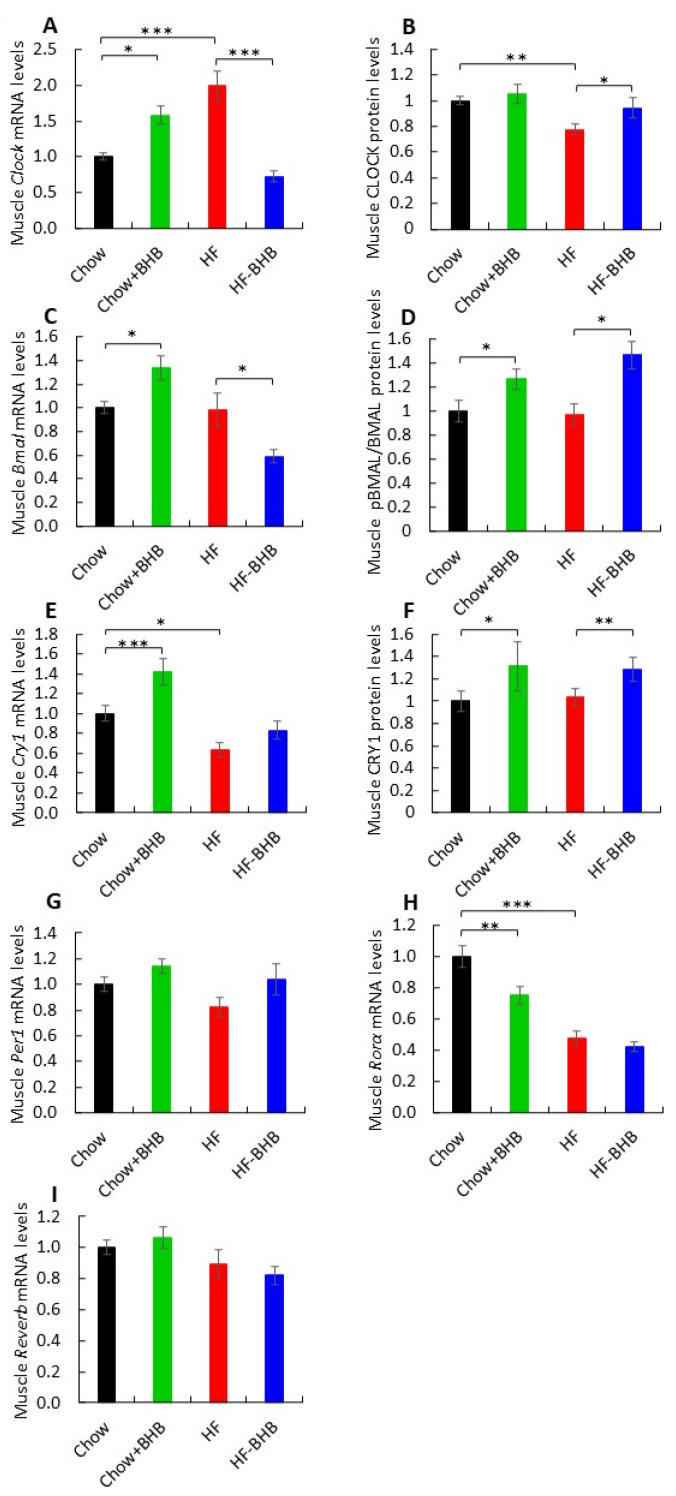
Effect of BHB on muscle circadian rhythms. (**A**) Average daily *Clock* mRNA levels in muscle tissue. (**B**) Average daily CLOCK levels in muscle tissue. (**C**) Average daily *Bmal1* mRNA levels in muscle tissue. (**D**) Average daily BMAL1 levels in muscle tissue. (**E**) Average daily *Cry1* mRNA levels in muscle tissue. (**F**) Average daily CRY1 levels in muscle tissue. (**G**) Average daily *Per1* mRNA levels in muscle tissue. (**H**) Average daily *Rorα* mRNA levels in muscle tissue. (**I**) Average daily *Rev-erbα* mRNA levels in muscle tissue. Circadian parameters (amplitude, phase) were derived using CircWave harmonic regression analysis. Data are presented as mean ± SEM. * *p* < 0.05, ** *p* < 0.01, *** *p* < 0.0001.

In skeletal muscle, BHB supplementation increased *Clock* mRNA expression in Chow-fed mice (*p* < 0.05) but reduced it in HF-fed mice (*p* < 0.0001) (Figure 6A). CLOCK protein levels were significantly lower in HF mice compared with Chow-fed mice (*p* < 0.01) (Figure 6B, Supplementary Figure S6). Although rhythmicity was preserved, amplitude was reduced under HF conditions (Supplementary Figure S7B). *Bmal1* mRNA levels were elevated in Chow + BHB mice compared with Chow animals (*p* < 0.05) but reduced in HF + BHB mice compared with HF animals (*p* < 0.05) (Figure 6C). Notably, both BHB-treated groups exhibited increased pBMAL1/BMAL1 ratios compared with Chow or HF animals (*p* < 0.05) (Figure 6D, Supplementary Figure S6). *Cry1* mRNA and protein levels were significantly increased by BHB compared with Chow or HF animals (*p* < 0.01) (Figure 6E,F, Supplementary Figure S6), accompanied by a phase advance in rhythmic expression (Supplementary Figure S7C). *Per1* mRNA levels were similar among groups (Figure 6G) but peaked at higher levels in HF + BHB animals (Supplementary Figure S7E). *Rorα* expression was reduced by both HF feeding and BHB supplementation compared with Chow animals (*p* < 0.0001) (Figure 6H), although rhythmicity was maintained (Supplementary Figure S7F). *Rev-erbα* expression did not differ significantly among groups (Figure 6I) but displayed altered rhythmicity in HF mice (Supplementary Figure S7G). Finally, *Myogenin* expression followed a diurnal pattern in all groups, with BHB-treated mice exhibiting higher peak levels (*p* < 0.01) (Figure 3H,I) and modified rhythmic profiles (Supplementary Figure S7A).

### 3.7. Effect of BHB on Adipose Tissue Circadian Rhythms

**Figure 7:**
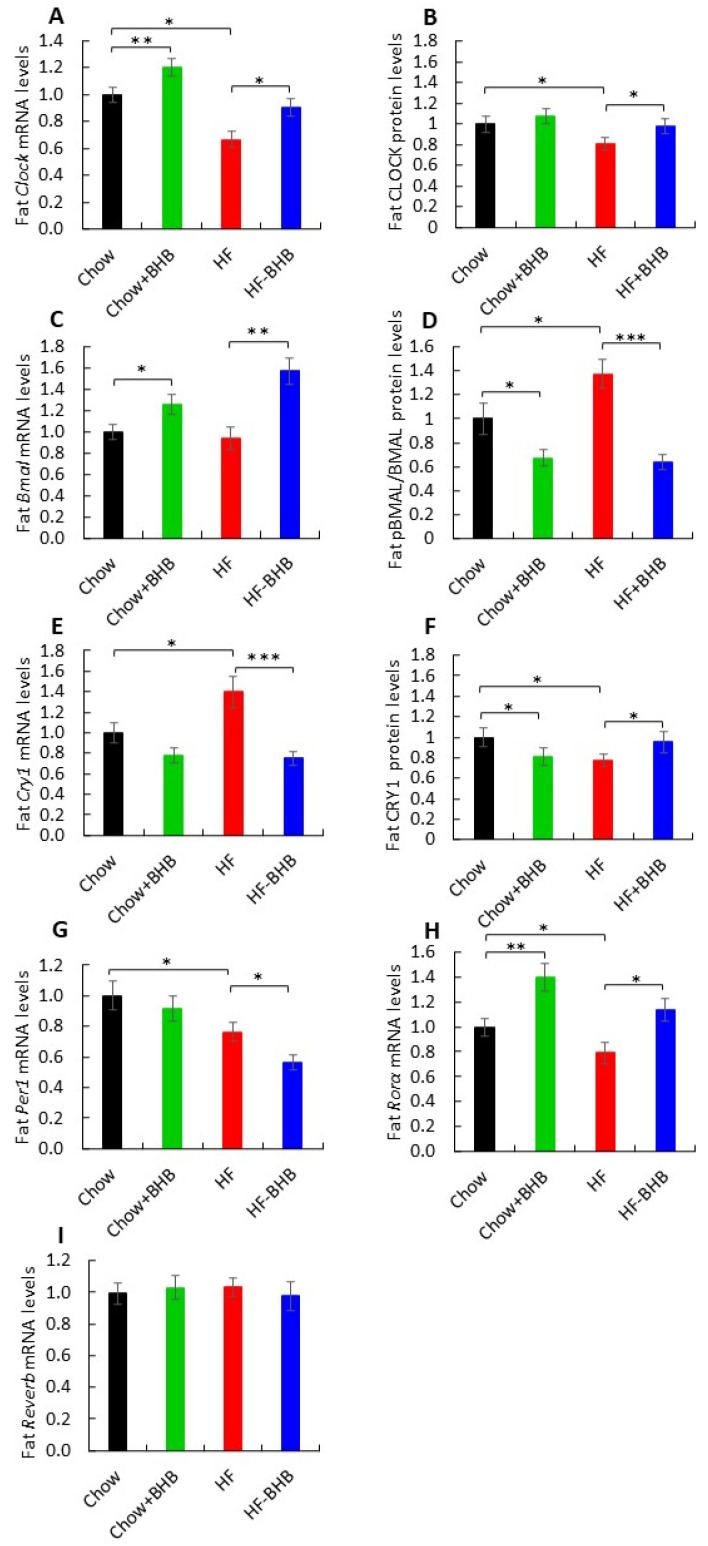
Effect of BHB on adipose tissue circadian rhythms. (**A**) Average daily *Clock* mRNA levels in adipose tissue. (**B**) Average daily CLOCK levels in adipose tissue. (**C**) Average daily *Bmal1* mRNA levels in adipose tissue. (**D**) Average daily BMAL1 levels in adipose tissue. (**E**) Average daily *Cry1* mRNA levels in adipose tissue. (**F**) Average daily CRY1 levels in adipose tissue. (**G**) Average daily *Per1* mRNA levels in adipose tissue. (**H**) Average daily *Rorα* mRNA levels in adipose tissue. (**I**) Average daily *Rev-erbα* mRNA levels in adipose tissue. Circadian parameters (amplitude, phase) were derived using CircWave harmonic regression analysis. Data are presented as mean ± SEM. * *p* < 0.05, ** *p* < 0.01, *** *p* < 0.0001.

In adipose tissue, HF feeding significantly reduced *Clock* mRNA and protein levels compared with Chow animals (*p* < 0.01 and *p* < 0.05) (Figure 7A,B, Supplementary Figure S8), whereas BHB supplementation restored their expression (*p* < 0.05). BHB increased *Bmal1* mRNA levels under both chow and HF conditions (*p* < 0.05 and *p* < 0.01) (Figure 7C). The pBMAL1/BMAL1 ratio, a marker of clock protein turnover, was elevated by HF feeding (*p* < 0.05) (Figure 7D, Supplementary Figure S8) and suppressed by BHB treatment in both dietary conditions (*p* < 0.05 and *p* < 0.0001). *Cry1* mRNA levels were increased in HF mice compared with Chow animals and reduced in BHB-treated groups (*p* < 0.05 and *p* < 0.0001) (Figure 7E). In contrast, CRY1 protein levels were decreased by HF feeding compared with Chow animals but significantly increased with BHB supplementation. In chow-fed mice, BHB reduced CRY1 protein levels (*p* < 0.05) (Figure 7F, Supplementary Figure S8). *Per1* mRNA expression was decreased in HF mice compared with Chow animals and further reduced by BHB (*p* < 0.05) (Figure 7G). *Rorα* expression was suppressed by HF feeding compared with Chow animals (*p* < 0.05) but significantly restored by BHB (*p* < 0.05) (Figure 7H).

*Rev-erbα* mRNA levels did not differ among the groups (*p* > 0.05) (Figure 7I). Across the 24-h cycle, HF feeding flattened or shifted peak expression of several clock genes, including *Per1*, *Clock*, and *Bmal1*. BHB supplementation modulated both the amplitude and phase of these oscillations, particularly for *Per1* and *Rorα*, suggesting partial restoration of peripheral clock rhythmicity. Collectively, HF feeding suppressed key positive-arm clock components in adipose tissue, an effect that was mitigated by BHB supplementation (Supplementary Figure S9).

## 4. Discussion

The present study extends previous findings by providing a comprehensive 24-h, multi-tissue analysis of metabolic and circadian responses to BHB under high-fat diet conditions. While prior studies have reported individual effects of BHB on metabolic signaling pathways, the integration of circadian profiling across multiple peripheral tissues represents a novel contribution. Our results suggest that BHB exerts multi-tissue metabolic and circadian regulatory effects under both chow and HF dietary conditions. HF feeding induced classical metabolic disturbances, including increased adiposity, reduced AMPK activation, enhanced AKT signaling, elevated inflammatory markers and disrupted peripheral clock gene oscillations. BHB supplementation partially reversed many of these alterations, particularly in adipose tissue and skeletal muscle, suggesting that ketone signaling modulates both metabolic and circadian homeostasis. However, these effects should be interpreted in the context of the significant reduction in food intake observed in the HF + BHB group, which may independently influence metabolic and circadian pathways. Reduced caloric intake is well known to activate AMPK, suppress mTOR signaling and modulate inflammatory and circadian pathways [21]. Therefore, some of the observed effects in the HF + BHB group may be partially attributable to reduced energy intake rather than direct effects of BHB alone.

HF feeding induced clear hepatic metabolic dysregulation, characterized by reduced AMPK activation and increased AKT signaling, alongside elevated NF-κB expression. Suppression of hepatic AMPK is a well-established feature of nutrient excess and insulin resistance, and its reduction promotes anabolic processes while impairing fatty acid oxidation [22,23]. The increased pAKT/AKT ratio in HF-fed mice likely reflects compensatory hyperinsulinemia and altered insulin signaling dynamics commonly observed in early diet-induced obesity. BHB supplementation attenuated HF-induced AKT hyperactivation and reduced NF-κB levels, suggesting improved inflammatory and metabolic signaling, as has been demonstrated [11,18]. The reduction in pmTOR/mTOR in Chow + BHB mice further supports a shift toward a more energy-conserving state, consistent with the known interaction between AMPK and mTOR pathways [24]. Although hepatic ACC and P70S6K signaling were not markedly altered, the overall pattern indicates that BHB is associated with changes in metabolic signaling pathways, primarily through anti-inflammatory actions and modulation of insulin-related signaling rather than robust activation of lipid oxidation pathways.

Skeletal muscle exhibited a pronounced metabolic response to BHB. HF feeding reduced AMPK activity, whereas BHB supplementation significantly increased the pAMPK/AMPK ratio and enhanced ACC phosphorylation, indicating stimulation of fatty acid oxidation. The observed increase in AMPK activation may reflect both BHB-related signaling and the effects of reduced caloric intake. Previous studies using caloric restriction paradigms have demonstrated similar activation of AMPK and improvements in metabolic flexibility, supporting this interpretation [21]. AMPK activation in muscle is central to improving metabolic flexibility and mitochondrial function [25]. Restoration of AMPK phosphorylation by BHB, especially in skeletal muscle and adipose tissue, aligns with growing evidence that ketone bodies act as signaling metabolites rather than merely oxidative substrates [9]. BHB has been shown to activate AMPK indirectly through alterations in cellular energy charge and redox state [12], which may explain the improved ACC phosphorylation and suppression of anabolic mTOR signaling observed here. The reduction in pmTOR/mTOR and pP70S6K/P70S6K ratios in BHB-treated groups further supports a shift toward a catabolic, energy-conserving phenotype [10]. This shift may reflect a substrate transition toward lipid and ketone utilization. In addition, the increase in myogenin expression further indicates that BHB promotes a transcriptional environment supportive of muscle remodeling and metabolic adaptation. Collectively, these findings suggest that skeletal muscle is particularly sensitive to BHB-induced metabolic reprogramming, likely due to its high oxidative capacity and role as a major site of ketone utilization.

Adipose tissue displayed substantial HF-induced metabolic impairment, including reduced AMPK activation and increased ACC, AKT, mTOR, and inflammatory signaling. These alterations are consistent with enhanced lipogenesis, suppressed lipid oxidation and chronic inflammation characteristic of obese adipose tissue [26]. BHB supplementation restored AMPK activation and reduced pACC/ACC, pAKT/AKT, pmTOR/mTOR and pP70S6K/P70S6K ratios, indicating suppression of anabolic and lipogenic pathways. The reduction in FASN levels with BHB further supports decreased lipogenesis. Importantly, NF-κB levels were markedly reduced by BHB under both chow and HF conditions, reinforcing its anti-inflammatory role [17]. Changes observed in adipose tissue and inflammatory markers may be influenced by the combined effects of BHB supplementation and reduced caloric intake. Because adipose tissue inflammation directly contributes to systemic insulin resistance, these findings suggest that BHB-mediated metabolic improvements in adipose tissue may have whole-body consequences.

When integrating these tissue-specific responses, a coordinated pattern emerges. HF feeding drives a systemic shift toward anabolic, inflammatory and metabolically inflexible states, characterized by reduced AMPK signaling and enhanced AKT/mTOR activity. BHB supplementation counteracts many of these effects in a tissue-dependent manner. In skeletal muscle and adipose tissue, BHB robustly activates AMPK and suppresses mTOR signaling, promoting a catabolic and oxidative phenotype. In the liver, BHB primarily attenuates inflammatory and insulin-related signaling abnormalities rather than strongly activating AMPK. Across tissues, the suppression of NF-κB suggests that reduced inflammation may be a unifying mechanism underlying improved metabolic signaling [17]. Inflammation is a key driver of metabolic dysfunction in obesity. The marked increase in NF-κB expression in HF-fed mice and its reversal by BHB are consistent with previous findings demonstrating that BHB suppresses inflammatory signaling, including inhibition of the NLRP3 inflammasome and NF-κB activity [17,18]. Thus, BHB supplementation may mitigate HF-induced metabolic impairment in part through anti-inflammatory mechanisms.

A central finding of this study is the disruption of peripheral circadian rhythms by HF feeding and their partial restoration by BHB. Obesity and HF diets are known to blunt the amplitude and alter the phase of clock gene expression in metabolic tissues [5,6,7]. Here, HF feeding reduced the expression and/or rhythmicity of key positive-arm clock components, including *Clock* and *Bmal1*, particularly in adipose tissue. These findings are consistent with reports that metabolic overload dampens peripheral clock oscillations and desynchronizes tissue-specific rhythms [27]. BHB supplementation partially restored *Clock* and *Bmal1* expression and normalized BMAL1 phosphorylation in adipose tissue, suggesting improved clock protein stability and turnover. Importantly, BHB modulated both amplitude and phase of oscillations in genes such as *Per1* and *Rorα*. Interestingly, the effects of BHB were tissue-specific. While adipose tissue showed robust restoration of positive-arm clock components, hepatic responses were more modest and included phase shifts rather than full amplitude recovery. This differential sensitivity may reflect tissue-specific ketone utilization rates or distinct clock-metabolism coupling mechanisms. The observed discrepancies between mRNA and protein levels for certain clock components may be explained by post-transcriptional regulation, differences in protein stability, or temporal delays between transcription and translation [28].

The interaction between cellular metabolism and the molecular clock is bidirectional: AMPK directly phosphorylates CRY proteins, promoting their degradation and thereby resetting clock timing [29]. Moreover, ketone bodies have been shown to function as epigenetic regulators via histone deacetylase (HDAC) inhibition [9,30], which may influence circadian gene transcription. Therefore, the clock-modulating effects of BHB observed here may result from combined AMPK activation, altered redox signaling and epigenetic regulation. In addition, BHB enhanced muscle myogenin expression and modified circadian gene rhythmicity, suggesting coordinated regulation of myogenic and clock pathways. Muscle clock genes are tightly linked to metabolic flexibility and mitochondrial function [31]. Thus, BHB-mediated improvements in muscle AMPK signaling and clock amplitude may contribute to enhanced metabolic adaptability.

An important limitation of this study is the significant reduction in food intake observed in the HF + BHB group. Reduced caloric intake alone can influence metabolic signaling pathways, including activation of AMPK, suppression of mTOR signaling and modulation of inflammatory pathways. Therefore, it is difficult to fully distinguish between direct effects of BHB and secondary effects due to reduced energy intake. Future studies employing pair-feeding designs will be necessary to isolate the specific contribution of BHB. Another limitation is that circulating BHB levels were assessed only under fasting conditions at a single time point. Although BHB supplementation was administered in the diet, circulating BHB levels were not elevated under fasting conditions at the time of measurement. This limits our ability to confirm direct systemic effects of BHB in the present dataset. Several alternative explanations should be considered. First, BHB levels may increase transiently in the postprandial state and therefore not be captured in fasting measurements. Second, BHB may be rapidly taken up and metabolized by peripheral tissues, resulting in tissue-specific effects without sustained elevation in circulation. Third, some observed effects may be indirectly mediated through changes in feeding behavior and energy balance. Thus, the observed metabolic and circadian changes may reflect a combination of direct and indirect mechanisms. Continuous or time-resolved measurements would be required to better characterize systemic exposure. While parallel changes in metabolic signaling and circadian gene expression were observed, the present study does not establish a causal relationship between these processes. The findings should therefore be interpreted as correlative.

## 5. Conclusions

**Figure 8:**
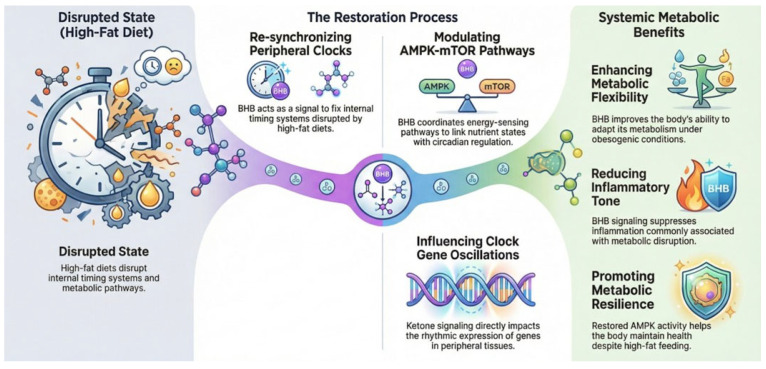
BHB as a metabolic bridge linking high-fat diet-induced circadian disruption to systemic metabolic restoration. Schematic illustration of the proposed mechanism by which BHB counteracts HF diet-induced metabolic and circadian disturbances. **Left panel**: HF feeding disrupts peripheral clock synchronization and metabolic signaling pathways, leading to impaired energy sensing, increased inflammatory tone, and altered clock gene oscillations. **Middle panel**: BHB acts as a signaling metabolite that re-synchronizes peripheral clocks, coordinates nutrient-sensing pathways through modulation of AMPK and mTOR signaling, and directly influences rhythmic clock gene expression. **Right panel**: These coordinated actions enhance metabolic flexibility, reduce inflammation and promote metabolic resilience under obesogenic conditions (generated via NotebookLM application at https://notebooklm.google.com/ accessed on 6 April 2026).

While previous studies have examined the effects of HF diets or BHB supplementation independently, the present study uniquely integrates 24-h circadian profiling across multiple metabolic tissues. This approach allows the identification of tissue-specific patterns of metabolic and circadian modulation that have not been fully characterized previously. Our findings support the concept that BHB may act as a systemic metabolic signal that enhances energy sensing, reduces inflammatory tone and promotes metabolic flexibility (Figure 8). The coordinated modulation of AMPK-mTOR pathways and peripheral clock machinery suggests that ketone signaling may serve as a mechanistic link between nutrient state, circadian regulation and tissue-specific metabolic adaptation. These findings support the concept that BHB may function as a metabolic signaling molecule capable of partially re-synchronizing peripheral circadian clocks disrupted by HF feeding. By partially restoring AMPK activity, suppressing inflammatory signaling, modulating mTOR pathways and influencing clock gene oscillations, BHB may promote metabolic resilience under obesogenic conditions. Further studies are needed to determine whether these clock-restorative effects translate into long-term improvements in insulin sensitivity and systemic metabolic health, and whether timing of BHB administration relative to circadian phase enhances its therapeutic efficacy.

## Data Availability

The datasets generated during and/or analyzed during the current study are available from the corresponding author upon reasonable request.

## Supplementary Materials

The following supporting information can be downloaded at: https://www.mdpi.com/article/10.3390/foods15081305/s1, Figure S1: Western blot protein analysis of the effect of HF and BHB supplementation on liver tissue metabolism; Figure S2: Western blot protein analysis of the effect of HF and BHB supplementation on muscle tissue metabolism; Figure S3: Western blot protein analysis of the effect of HF and BHB supplementation on adipose tissue metabolism; Figure S4: Western blot protein analysis of the effect of HF and BHB supplementation on the circadian clock in liver tissue; Figure S5: Effect of BHB on the circadian clock oscillation in liver tissue; Figure S6: Western blot protein analysis of the effect of HF and BHB supplementation on the circadian clock in muscle tissue; Figure S7: Effect of BHB on the circadian clock oscillation in muscle tissue; Figure S8: Western blot protein analysis of the effect of HF and BHB supplementation on the circadian clock in adipose tissue; Figure S9: Effect of BHB on the circadian clock oscillation in adipose tissue.

## Abbreviations

The following abbreviations are used in this manuscript:

AKT (PKB)	Protein kinase B
BMAL1	Brain and muscle Arnt-like protein-1
CLOCK	Circadian locomotor output cycles kaput
CRY	Cryptochrome
BHB	β-Hydroxybutyrate
mTOR	Mammalian target of rapamycin
PER	Period
REV-ERB	Reverse *Erb*
ROR	Retinoic acid-related orphan receptor
SCN	Suprachiasmatic nuclei
