# Supplementary material for: β-Hydroxybutyrate Modulates Metabolic Signaling and Partially Restores Peripheral Circadian Rhythms in High-Fat Diet-Fed Mice
Source: Foods. 2026 Apr 9;15(8):1305. doi: 10.3390/foods15081305 (PMC13115401; doi:10.3390/foods15081305)
Supplement: Supplementary file 1 [file foods-15-01305-s001.zip › foods-4217839-supplementary.pptx]

## Slide 1
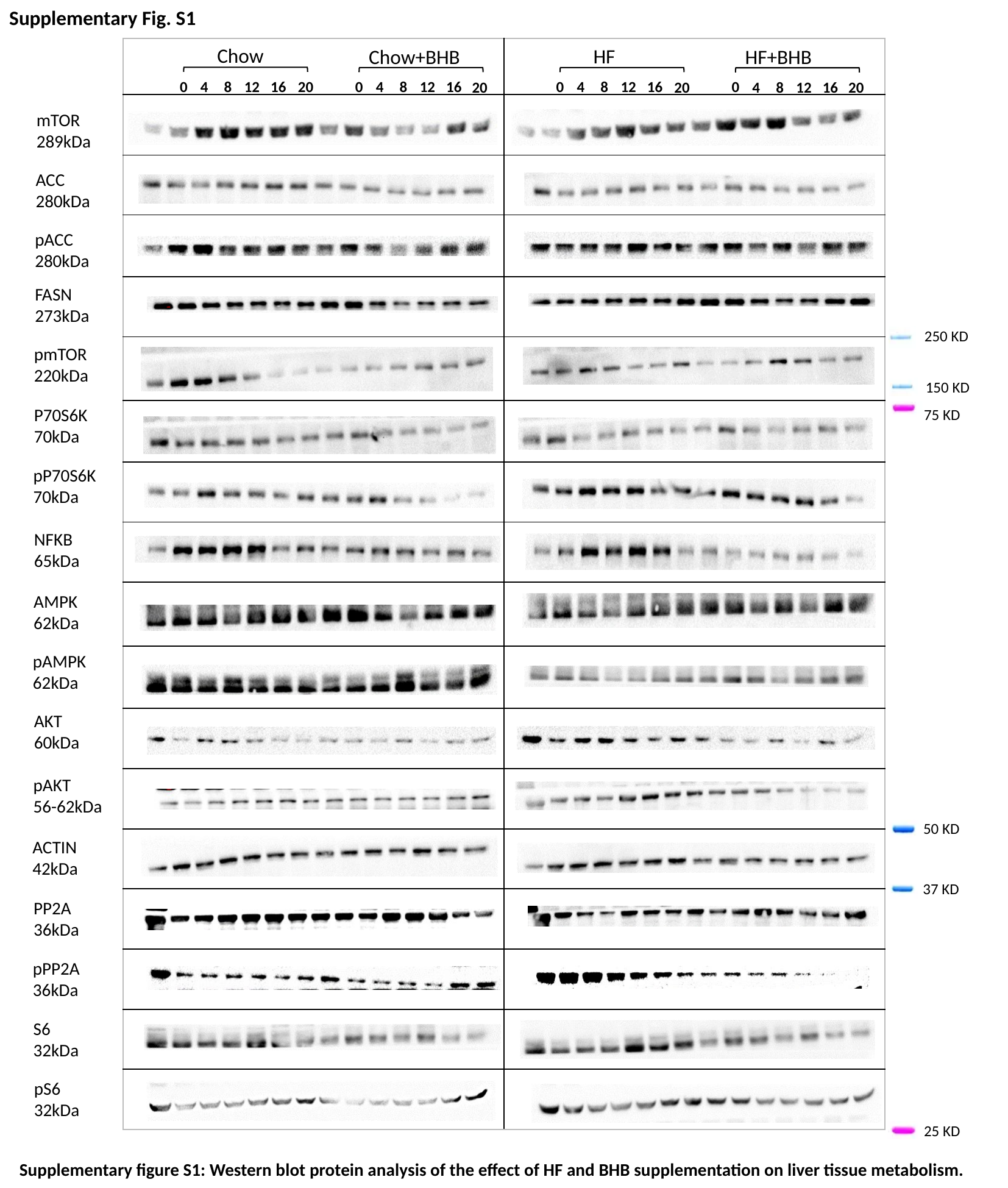

Supplementary Fig. S1
| | |
| --- | --- |
| | |
| | |
| | |
| | |
| | |
| | |
| | |
| | |
| | |
| | |
| | |
| | |
| | |
| | |
| | |
| | |
| | |
Chow
HF
Chow+BHB
HF+BHB
0
8
12
16
4
0
8
12
16
4
0
8
12
16
4
0
8
12
16
4
20
20
20
20
mTOR 289kDa
ACC 280kDa
pACC
280kDa
FASN 273kDa
250 KD
pmTOR
220kDa
150 KD
P70S6K
70kDa
75 KD
pP70S6K
70kDa
NFKB
65kDa
AMPK
62kDa
pAMPK
62kDa
AKT 60kDa
pAKT
56-62kDa
50 KD
ACTIN
42kDa
37 KD
PP2A
36kDa
pPP2A
36kDa
S6
32kDa
pS6
32kDa
25 KD
Supplementary figure S1: Western blot protein analysis of the effect of HF and BHB supplementation on liver tissue metabolism.

## Slide 2
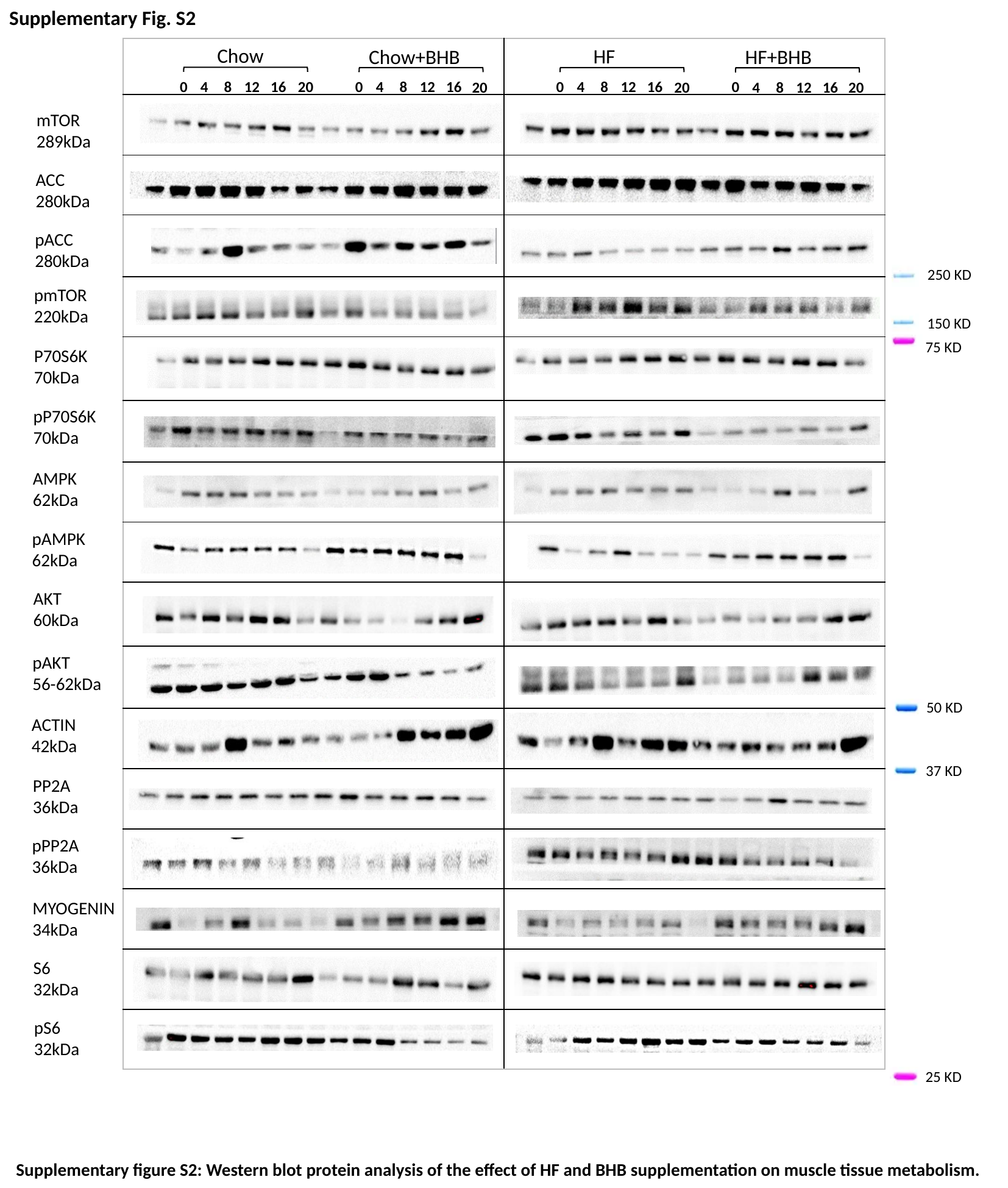

Supplementary Fig. S2
| | |
| --- | --- |
| | |
| | |
| | |
| | |
| | |
| | |
| | |
| | |
| | |
| | |
| | |
| | |
| | |
| | |
| | |
| | |
Chow
HF
Chow+BHB
HF+BHB
0
8
12
16
4
0
8
12
16
4
0
8
12
16
4
0
8
12
16
4
20
20
20
20
mTOR 289kDa
ACC 280kDa
pACC
280kDa
250 KD
pmTOR
220kDa
P70S6K
70kDa
pP70S6K
70kDa
150 KD
75 KD
AMPK
62kDa
pAMPK
62kDa
AKT 60kDa
pAKT
56-62kDa
ACTIN
42kDa
PP2A
36kDa
pPP2A
36kDa
50 KD
37 KD
MYOGENIN
34kDa
S6
32kDa
pS6
32kDa
25 KD
Supplementary figure S2: Western blot protein analysis of the effect of HF and BHB supplementation on muscle tissue metabolism.

## Slide 3
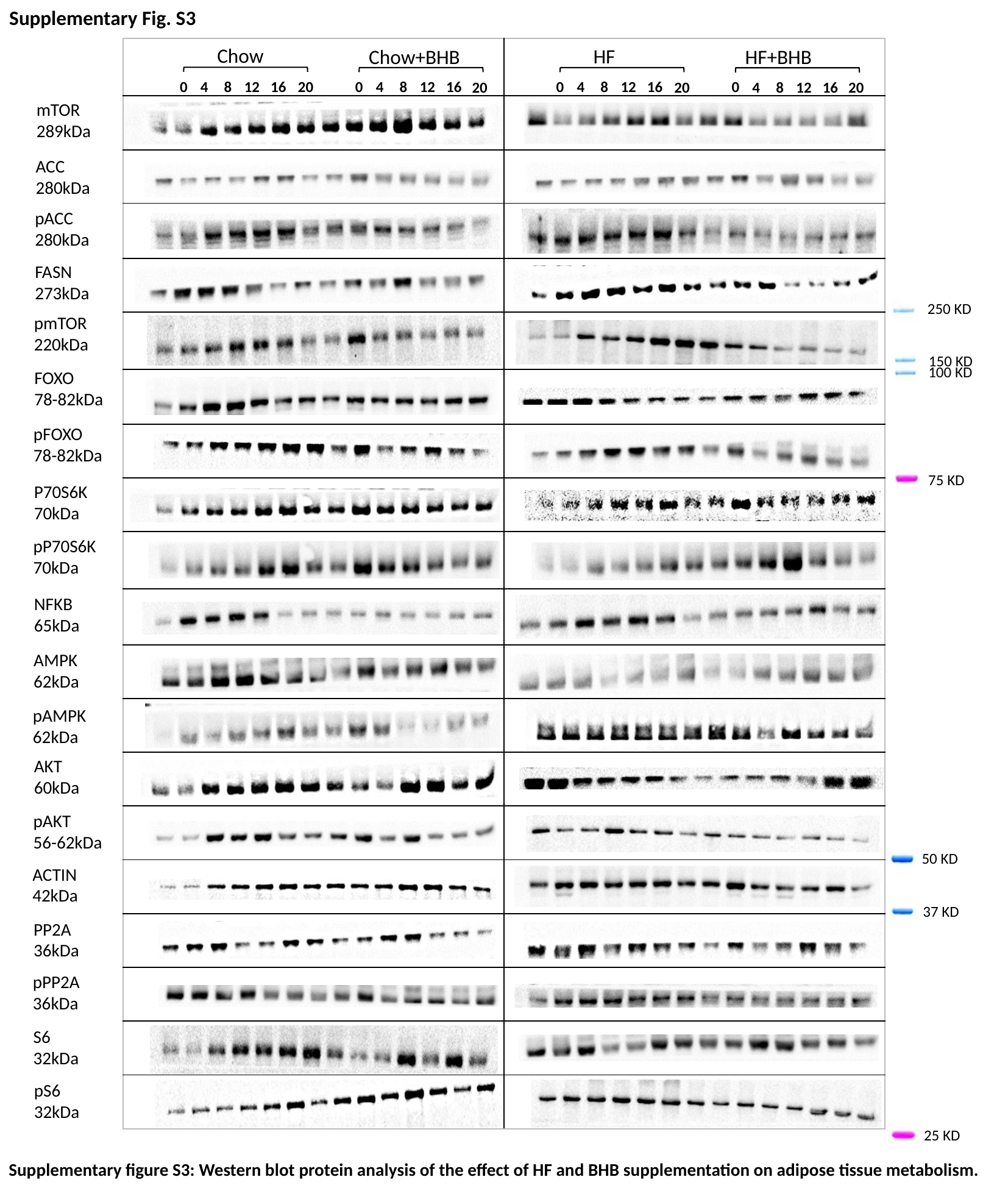

Supplementary Fig. S3
| | |
| --- | --- |
| | |
| | |
| | |
| | |
| | |
| | |
| | |
| | |
| | |
| | |
| | |
| | |
| | |
| | |
| | |
| | |
| | |
| | |
| | |
Chow
HF
Chow+BHB
HF+BHB
0
8
12
16
4
0
8
12
16
4
0
8
12
16
4
0
8
12
16
4
20
20
20
20
mTOR 289kDa
ACC 280kDa
pACC
280kDa
FASN 273kDa
250 KD
pmTOR
220kDa
150 KD
100 KD
FOXO
78-82kDa
pFOXO
78-82kDa
75 KD
P70S6K
70kDa
pP70S6K
70kDa
NFKB
65kDa
AMPK
62kDa
pAMPK
62kDa
AKT 60kDa
pAKT
56-62kDa
50 KD
ACTIN
42kDa
37 KD
PP2A
36kDa
pPP2A
36kDa
S6
32kDa
pS6
32kDa
25 KD
Supplementary figure S3: Western blot protein analysis of the effect of HF and BHB supplementation on adipose tissue metabolism.

## Slide 4
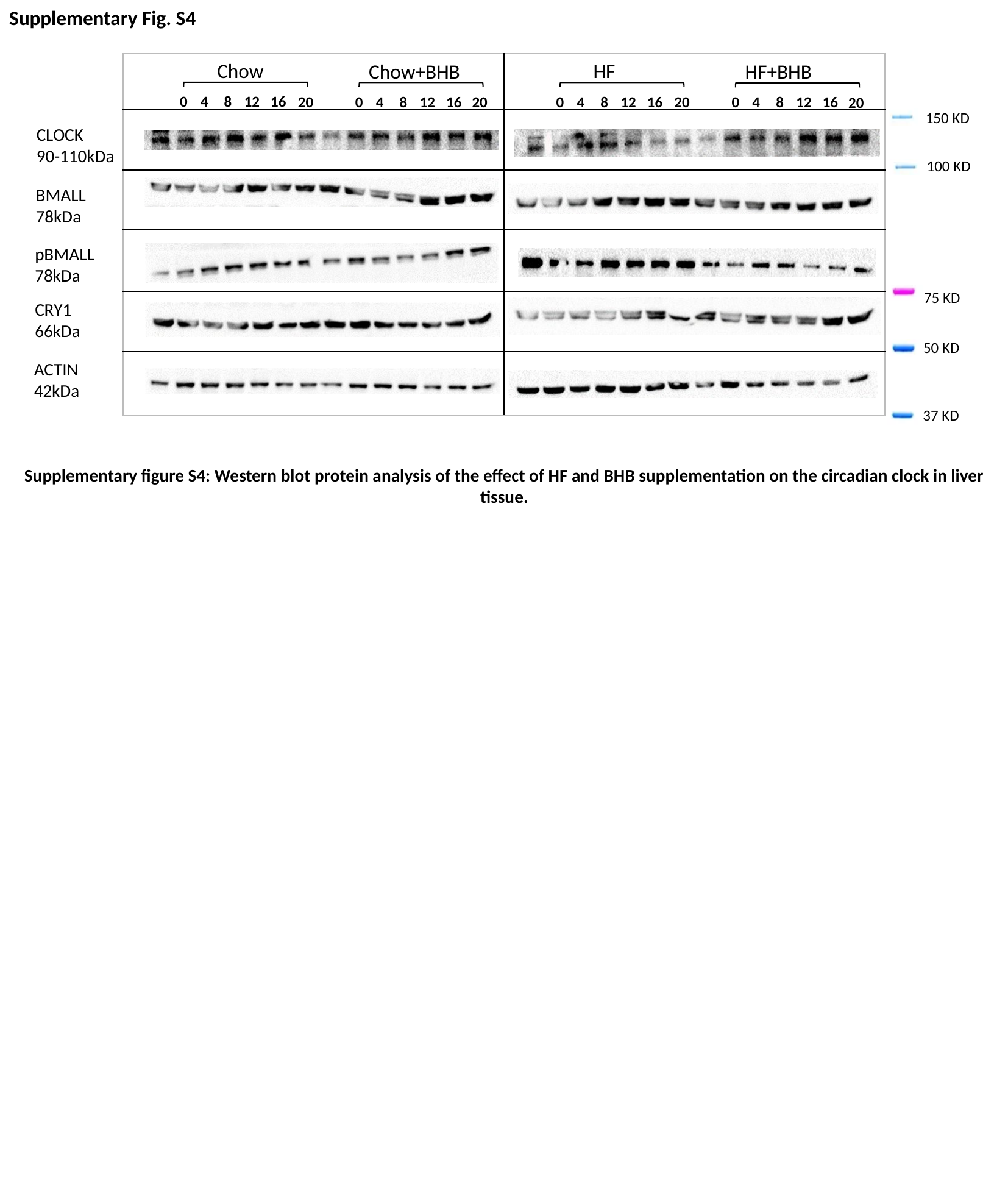

Supplementary Fig. S4
| | |
| --- | --- |
| | |
| | |
| | |
| | |
| | |
Chow
HF
Chow+BHB
HF+BHB
0
8
12
16
4
0
8
12
16
4
0
8
12
16
4
0
8
12
16
4
20
20
20
20
150 KD
CLOCK 90-110kDa
100 KD
BMALL 78kDa
pBMALL
78kDa
75 KD
CRY1 66kDa
50 KD
ACTIN
42kDa
37 KD
Supplementary figure S4: Western blot protein analysis of the effect of HF and BHB supplementation on the circadian clock in liver tissue.

## Slide 5
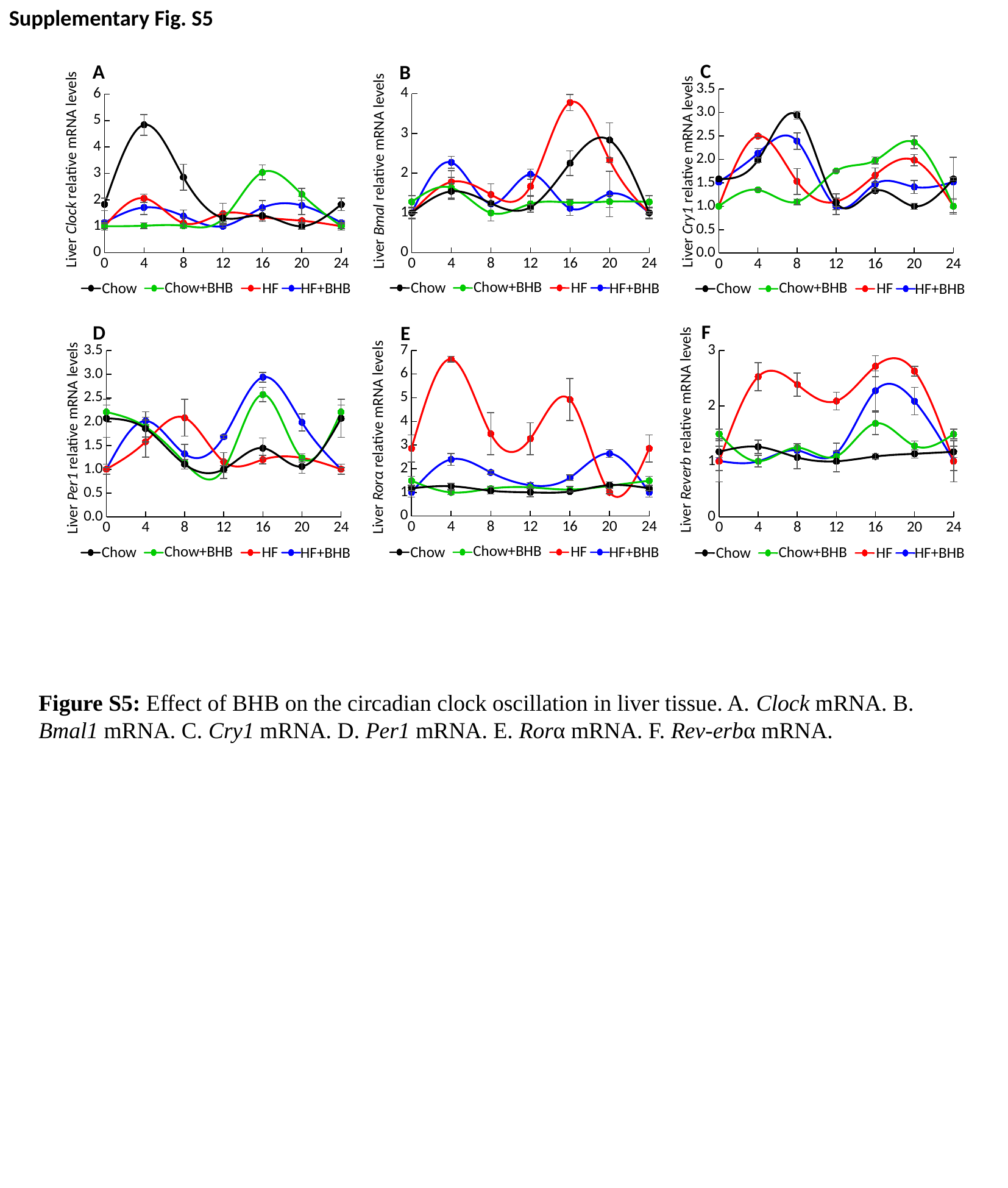

Supplementary Fig. S5
C
A
B
### Chart
| Category | Chow | Chow+BHB | HF | HF-BHB |
|---|---|---|---|---|
### Chart
| Category | Chow | Chow+BHB | HF | HF+BHB |
|---|---|---|---|---|
### Chart
| Category | Chow | Chow+BHB | HF | HF-BHB |
|---|---|---|---|---|Liver Clock relative mRNA levels
Liver Bmal relative mRNA levels
Liver Cry1 relative mRNA levels
Chow+BHB
HF
Chow
HF+BHB
Chow+BHB
HF
Chow
HF+BHB
Chow+BHB
HF
Chow
HF+BHB
F
D
E
### Chart
| Category | control | control+BHB | HF | HF+BHB |
|---|---|---|---|---|
### Chart
| Category | control | control+BHB | HF | HF+BHB |
|---|---|---|---|---|
### Chart
| Category | control | control+BHB | HF | HF+BHB |
|---|---|---|---|---|Liver Reverb relative mRNA levels
Liver Rorα relative mRNA levels
Liver Per1 relative mRNA levels
Chow+BHB
HF
Chow
HF+BHB
Chow+BHB
HF
Chow
HF+BHB
Chow+BHB
HF
Chow
HF+BHB
Figure S5: Effect of BHB on the circadian clock oscillation in liver tissue. A. Clock mRNA. B. Bmal1 mRNA. C. Cry1 mRNA. D. Per1 mRNA. E. Rorα mRNA. F. Rev-erbα mRNA.

## Slide 6
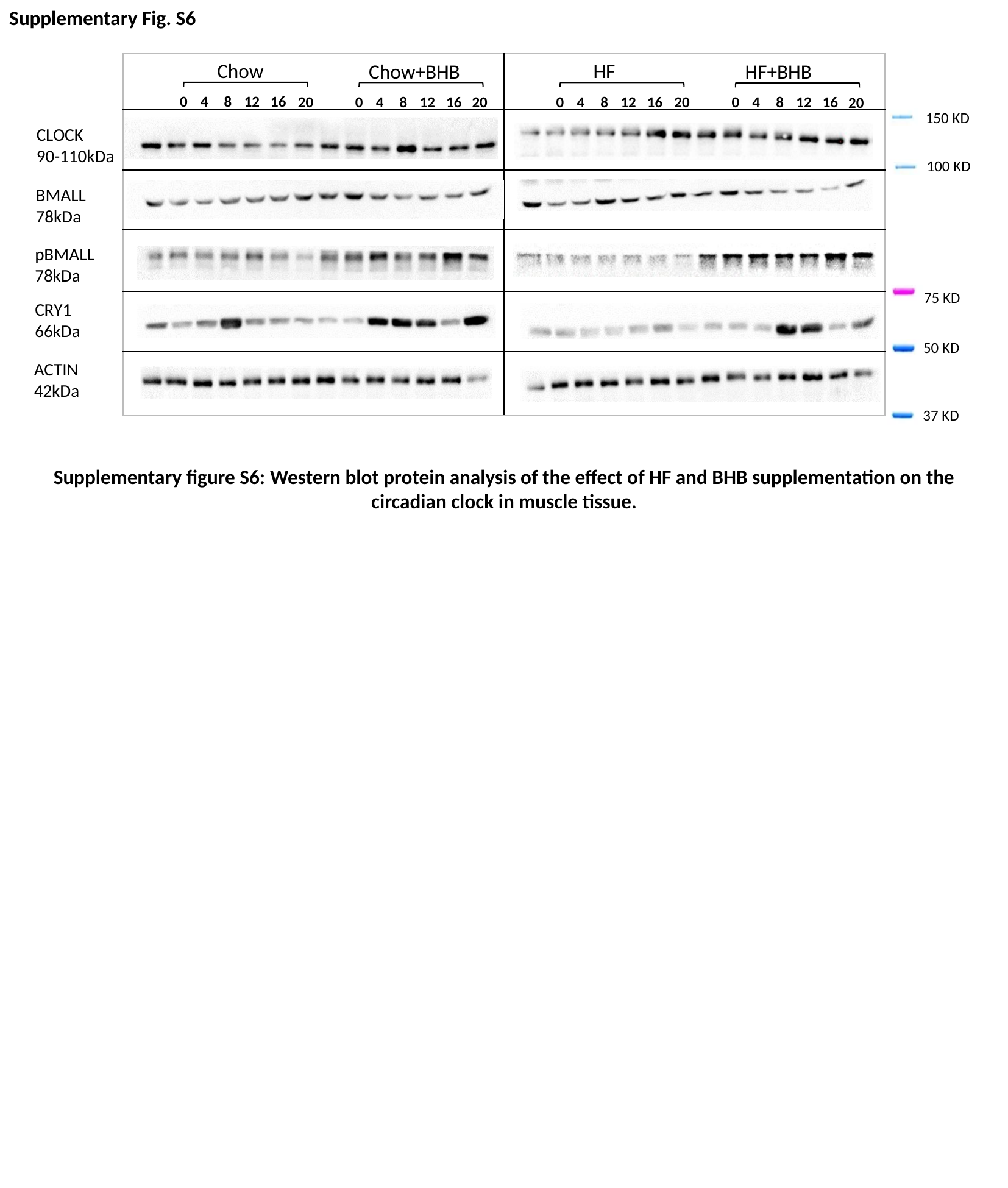

Supplementary Fig. S6
| | |
| --- | --- |
| | |
| | |
| | |
| | |
| | |
Chow
HF
Chow+BHB
HF+BHB
0
8
12
16
4
0
8
12
16
4
0
8
12
16
4
0
8
12
16
4
20
20
20
20
150 KD
CLOCK 90-110kDa
100 KD
BMALL 78kDa
pBMALL
78kDa
75 KD
CRY1 66kDa
50 KD
ACTIN
42kDa
37 KD
Supplementary figure S6: Western blot protein analysis of the effect of HF and BHB supplementation on the circadian clock in muscle tissue.

## Slide 7
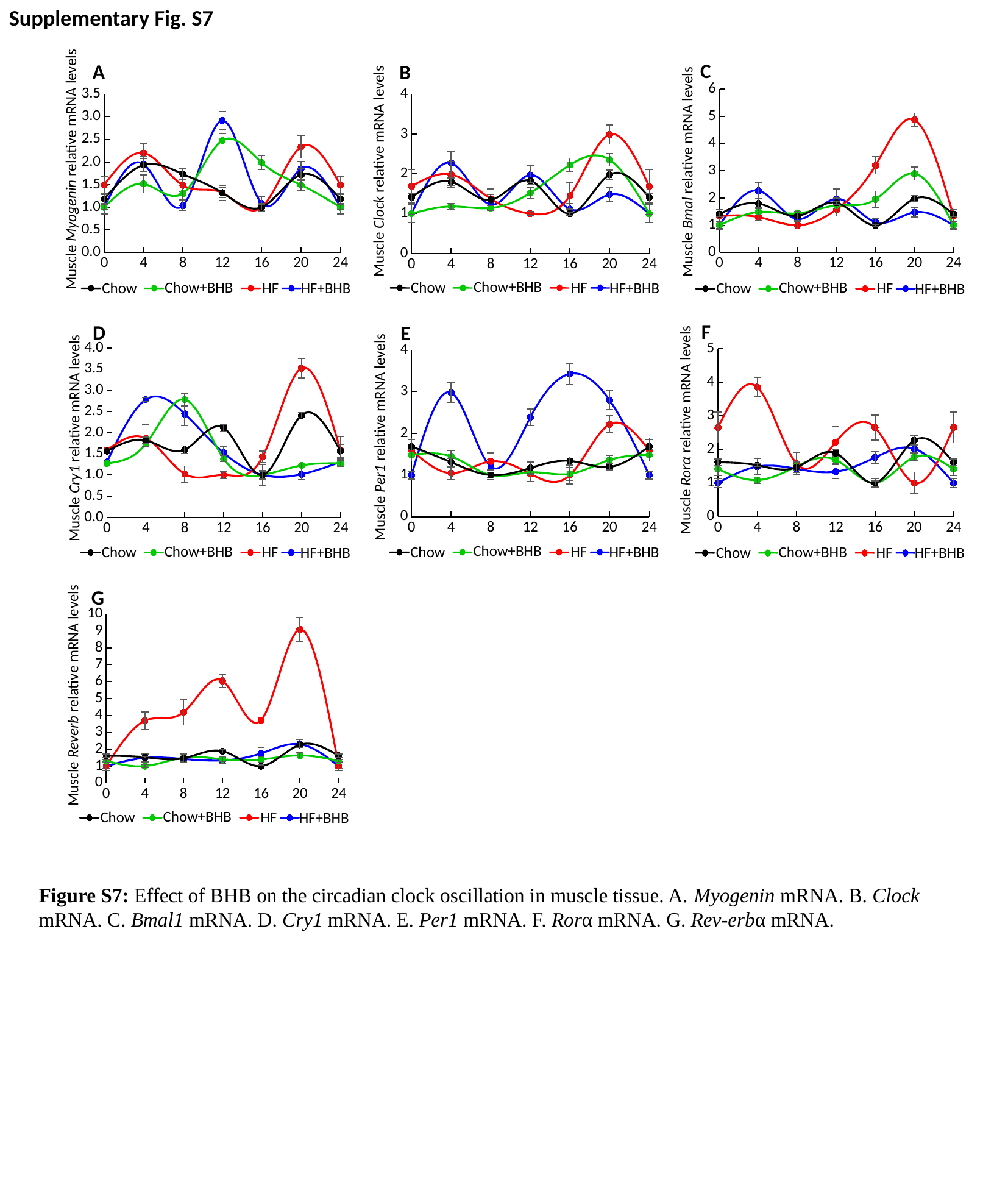

Supplementary Fig. S7
C
A
B
### Chart
| Category | control | control+BHB | HF | HF+BHB |
|---|---|---|---|---|
### Chart
| Category | control | control+BHB | HF | HF+BHB |
|---|---|---|---|---|
### Chart
| Category | control | control+BHB | HF | HF+BHB |
|---|---|---|---|---|Muscle Myogenin relative mRNA levels
Muscle Clock relative mRNA levels
Muscle Bmal relative mRNA levels
Chow+BHB
HF
Chow
HF+BHB
Chow+BHB
HF
Chow
HF+BHB
Chow+BHB
HF
Chow
HF+BHB
F
D
E
### Chart
| Category | control | control+BHB | HF | HF+BHB |
|---|---|---|---|---|
### Chart
| Category | control | control+BHB | HF | HF+BHB |
|---|---|---|---|---|
### Chart
| Category | control | control+BHB | HF | HF+BHB |
|---|---|---|---|---|Muscle Rorα relative mRNA levels
Muscle Per1 relative mRNA levels
Muscle Cry1 relative mRNA levels
Chow+BHB
HF
Chow
HF+BHB
Chow+BHB
HF
Chow
HF+BHB
Chow+BHB
HF
Chow
HF+BHB
G
### Chart
| Category | control | control+BHB | HF | HF+BHB |
|---|---|---|---|---|Muscle Reverb relative mRNA levels
Chow+BHB
HF
Chow
HF+BHB
Figure S7: Effect of BHB on the circadian clock oscillation in muscle tissue. A. Myogenin mRNA. B. Clock mRNA. C. Bmal1 mRNA. D. Cry1 mRNA. E. Per1 mRNA. F. Rorα mRNA. G. Rev-erbα mRNA.

## Slide 8
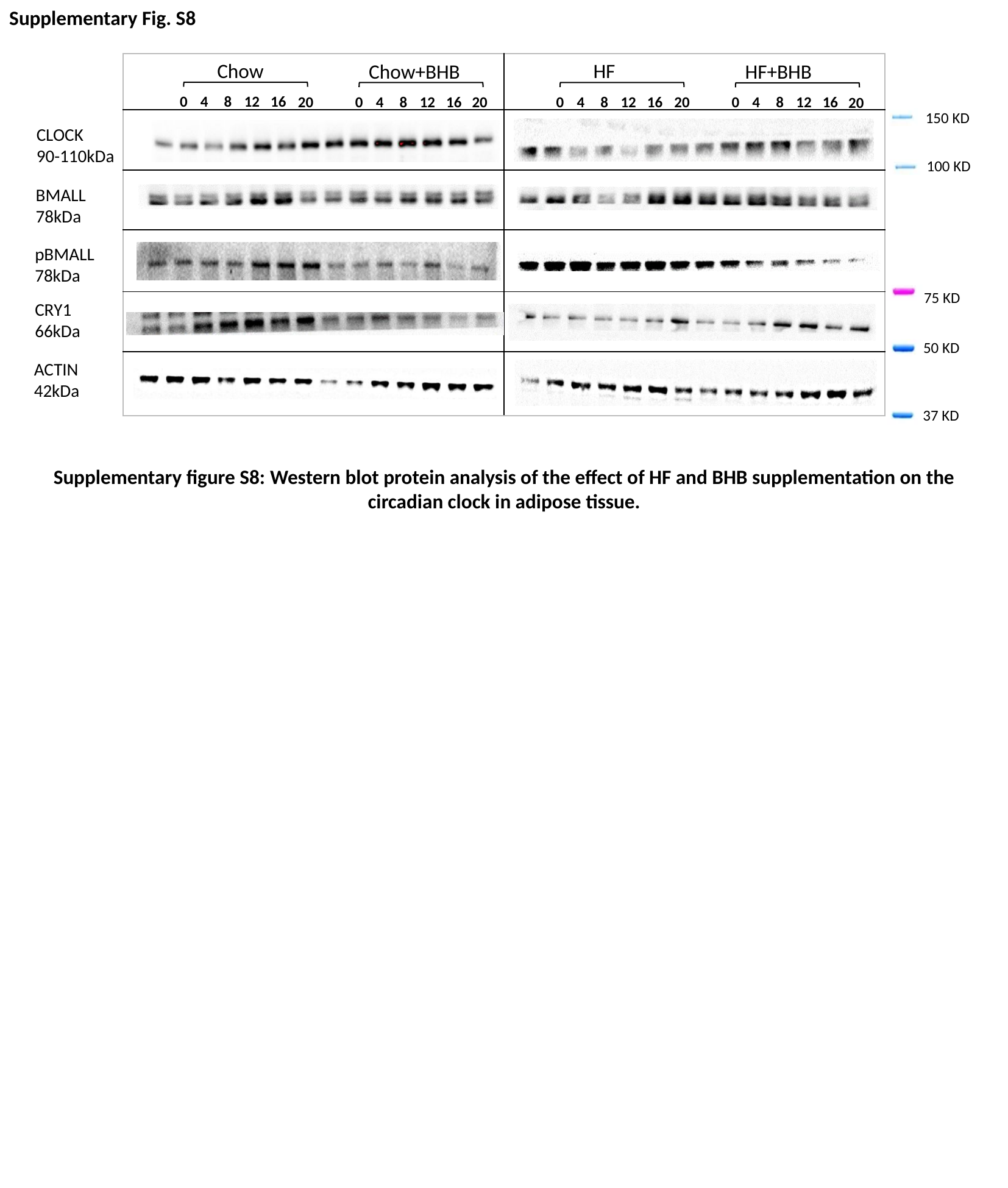

Supplementary Fig. S8
| | |
| --- | --- |
| | |
| | |
| | |
| | |
| | |
Chow
HF
Chow+BHB
HF+BHB
0
8
12
16
4
0
8
12
16
4
0
8
12
16
4
0
8
12
16
4
20
20
20
20
150 KD
CLOCK 90-110kDa
100 KD
BMALL 78kDa
pBMALL
78kDa
75 KD
CRY1 66kDa
50 KD
ACTIN
42kDa
37 KD
Supplementary figure S8: Western blot protein analysis of the effect of HF and BHB supplementation on the circadian clock in adipose tissue.

## Slide 9
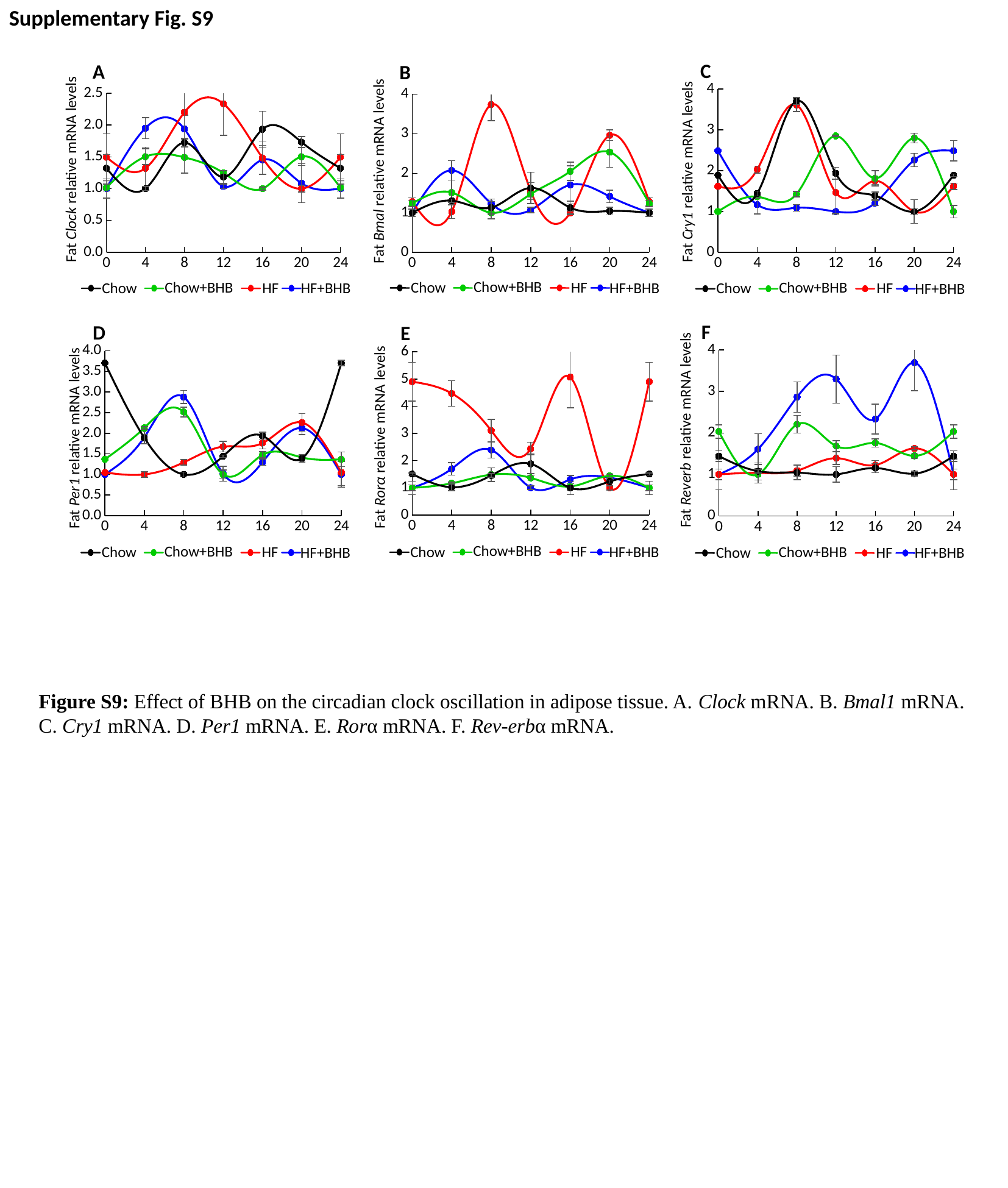

Supplementary Fig. S9
C
A
B
### Chart
| Category | control | control+BHB | HF | HF+BHB |
|---|---|---|---|---|
### Chart
| Category | control | control+BHB | HF | HF+BHB |
|---|---|---|---|---|
### Chart
| Category | control | control+BHB | HF | HF+BHB |
|---|---|---|---|---|Fat Clock relative mRNA levels
Fat Bmal relative mRNA levels
Fat Cry1 relative mRNA levels
Chow+BHB
HF
Chow
HF+BHB
Chow+BHB
HF
Chow
HF+BHB
Chow+BHB
HF
Chow
HF+BHB
F
D
E
### Chart
| Category | control | control+BHB | HF | HF+BHB |
|---|---|---|---|---|
### Chart
| Category | control | control+BHB | HF | HF+BHB |
|---|---|---|---|---|
### Chart
| Category | control | control+BHB | HF | HF+BHB |
|---|---|---|---|---|Fat Reverb relative mRNA levels
Fat Rorα relative mRNA levels
Fat Per1 relative mRNA levels
Chow+BHB
HF
Chow
HF+BHB
Chow+BHB
HF
Chow
HF+BHB
Chow+BHB
HF
Chow
HF+BHB
Figure S9: Effect of BHB on the circadian clock oscillation in adipose tissue. A. Clock mRNA. B. Bmal1 mRNA. C. Cry1 mRNA. D. Per1 mRNA. E. Rorα mRNA. F. Rev-erbα mRNA.
